# ATPase Mechanism of the 5′-3′ DNA Helicase, RecD2: EVIDENCE FOR A PRE-HYDROLYSIS CONFORMATION CHANGE*

**DOI:** 10.1074/jbc.M113.484667

**Published:** 2013-07-09

**Authors:** Christopher P. Toseland, Martin R. Webb

**Affiliations:** From the ‡ MRC National Institute for Medical Research, Mill Hill, London, NW7 1AA, United Kingdom and; § Institut für Zelluläre Physiologie and Center for NanoScience, Physiologisches Institut, Ludwig Maximilians Universität, Munich 80336, Germany

**Keywords:** ATPases, DNA Helicase, Enzyme Mechanisms, Fluorescence, Kinetics, Superfamily 1

## Abstract

The superfamily 1 helicase, RecD2, is a monomeric, bacterial enzyme with a role in DNA repair, but with 5′-3′ activity unlike most enzymes from this superfamily. Rate constants were determined for steps within the ATPase cycle of RecD2 in the presence of ssDNA. The fluorescent ATP analog, mantATP (2′(3′)-*O*-(*N*-methylanthraniloyl)ATP), was used throughout to provide a complete set of rate constants and determine the mechanism of the cycle for a single nucleotide species. Fluorescence stopped-flow measurements were used to determine rate constants for adenosine nucleotide binding and release, quenched-flow measurements were used for the hydrolytic cleavage step, and the fluorescent phosphate biosensor was used for phosphate release kinetics. Some rate constants could also be measured using the natural substrate, ATP, and these suggested a similar mechanism to that obtained with mantATP. The data show that a rearrangement linked to Mg^2+^ coordination, which occurs before the hydrolysis step, is rate-limiting in the cycle and that this step is greatly accelerated by bound DNA. This is also shown here for the PcrA 3′-5′ helicase and so may be a general mechanism governing superfamily 1 helicases. The mechanism accounts for the tight coupling between translocation and ATPase activity.

## Teaser

**Background:** Unlike most helicase superfamily 1 members, RecD2 moves with 5′-3′ direction, but its ATPase mechanism is unknown.

**Results:** A DNA-enhanced prehydrolysis conformation change limits the ATPase cycle.

**Conclusion:** The conformation change triggers hydrolysis, ensuring tight coupling between ATPase activity and DNA translocation for both RecD2 and PcrA.

**Significance:** Elucidating the mechanism of motor activity is crucial for understanding helicase function.

## Introduction

DNA helicases are motor proteins with essential roles in many aspects of DNA metabolism. The chemical energy of ATP hydrolysis drives DNA unwinding and translocation by the helicase. Work is presented here on the superfamily 1 (SF1)^3^ bacterial helicase, RecD2. SF1 enzymes can be divided on the basis of translocation: those that move with a 3′-5′ polarity, such as PcrA, UvrD, and Rep (SF1A), and those that move with a 5′-3′ polarity, including RecD2 (SF1B) (1). In general, SF1A helicases are better characterized from a biochemical and structural perspective than SF1B enzymes. SF1B helicases have important roles in eukaryotic and prokaryotic systems, with diverse functions such as maintenance of telomeres and ribosomal RNA genes, processing Okazaki fragments and RNA (2–5). The best characterized SF1B members are the T4 bacteriophage Dda and *Escherichia coli* RecD, which is one of the helicase motors in the *E. coli* RecBCD complex involved in DNA recombination. RecD2 belongs to the same family as RecD and was identified in *Deinococcus radiodurans* with a role in DNA repair. However, unlike *E. coli*, *D. radiodurans* does not contain genes for RecB and RecC (6), and so RecD2 may well not operate as part of a multihelicase complex.

High resolution, crystal structures showed RecD2 bound to single-stranded DNA (ssDNA) in the presence and absence of a non-hydrolyzable ATP analog, ADPNP (7). The ssDNA binds across the DNA binding domains in the same orientation with SF1A helicases, and so the 5′-3′ translocation is presumably brought about by the enzyme itself moving in the opposite direction to SF1A helicases. This is achieved by alternative binding of the ssDNA by the protein domains. In 3′–5′ helicases such as PcrA, when ATP binds, the DNA-protein interactions are strongest in domain 2A. However, with 5′–3′ helicases, domain 1A has the tightest hold on the DNA, and this triggers ssDNA being pulled in the opposite direction. Although RecD2 seems to be a poor helicase on double-stranded DNA (dsDNA), the translocation along ssDNA has been characterized, and the step size is one ATP per base moved (7). It is of considerable interest to see if these structural and polarity differences are reflected in the ATPase mechanism.

The mechanism will be compared with PcrA, one of the most characterized SF1A members. This helicase also hydrolyzes one ATP per base during both translocation and unwinding (8, 9) with a rate-limiting hydrolysis step (10). There must be tight coupling between hydrolytic cleavage of ATP and movement, as this ratio is maintained from ssDNA to dsDNA. The translocation rate is up to 10-fold higher than RepD-modulated unwinding (11), suggesting PcrA uses a passive mechanism (12, 13). In such a mechanism, thermal fluctuations affect the base pairing at the end of the dsDNA and so allows the helicase to translocate after fortuitous separation of the duplex. The tight coupling between hydrolysis and both translocation and unwinding prevents futile hydrolysis cycles, which can occur with a passive mechanism.

**FIGURE 1.**
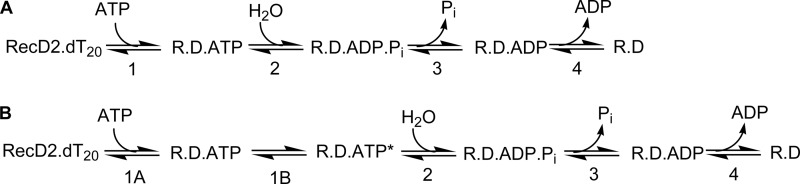
**ATPase reaction schemes.** *A*, shown is a minimal mechanism for ATP hydrolysis by RecD2 (*R*) with DNA (*D*). Steps are numbered, such that step *n* has forward and reverse rate constants, *k*_+_*_n_* and *k*_−_*_n_*, respectively, and equilibrium constant, *K_n_. B*, shown is a scheme including an ATP-bound conformation change (*step 1B*).

In this study the first full kinetic analysis of the ATPase cycle of a SF1B helicase is presented by investigating *D. radiodurans* RecD2. Individual steps of the cycle were measured using the fluorescent analog, mantATP, to provide a consistent view of the cycle, as some rate constants are not accessible with the natural ATP substrate. This experimental approach allowed almost all individual rate constants in the ATPase cycle (Fig. 1) to be defined and thereby to relate how the dynamics of the ATPase cycle to translocation events and structural changes. There is evidence for a rate-limiting conformation change occurring before the hydrolytic cleavage. The ATPase cycle is very similar to that of PcrA helicase, so that that directionality does not change the properties of the motor in this respect. Therefore, it is possible that there is a generic ATPase mechanism for SF1 members.

## EXPERIMENTAL PROCEDURES

### 

#### 

##### Materials

MantATP, mantADP, 3′-mant-2′-deoxyATP, and mantATPγS were synthesized from their parent nucleotides by a modification (14) of the method of Hiratsuka (15). Deac-aminoATP was synthesized as described (16, 17). Oligonucleotides were from Eurogentec Ltd (Southampton, UK). All other biochemical reagents were from Sigma.

RecD2 from *D. radiodurans* was expressed and purified as described previously (7). The construct had an N-terminal truncation lacking the first 150 amino acids, identical to the one used in the structural and biochemical assays published previously. Coumarin-labeled phosphate protein (MDCC-PBP) and rhodamine-labeled PBP (rhodamine-PBP) were prepared as described (18–20). *Bacillus stearothermophilus* PcrA was prepared as described previously (21). Mutants were generated using the QuikChange II site-directed mutagenesis kit (Stratagene) according to the manufacturer's instructions. Mutants were transformed into BL21 (DE3) pLysS cells (Novagen) and expressed as with the wild type protein (21).

##### Quench-flow Measurements

These were carried out using a HiTech RQF-63 apparatus using different length loops and flow rates to age reactions. Samples were quenched rapidly using 10% (v/v) perchloric acid and analyzed for the ratio of triphosphate to diphosphate by HPLC as described (10). The data shown are for a single set of measurements, but all were repeated to show similar results.

##### Optical Measurements

Stopped-flow fluorescence measurements used a HiTech SF61DX2 apparatus (TgK Scientific Ltd, Bradford-on-Avon, UK) with a mercury-xenon light source and HiTech Kinetic Studio 2 software. For MDCC-PBP and diethylaminocoumarin (deac)-nucleotide fluorescence, the excitation wavelength was 436, nm, and a 455-nm cut-off filter (Schott glass) was used to collect emitted light. Rhodamine-PBP was excited at 550 nm with a 570-nm cut-off filter (Schott glass) on the emission. For measurements using PBP, the signal was calibrated using known concentrations of inorganic phosphate (P_i_), as previously described. Mant fluorescence was excited at 366 nm, and a 400-nm cut-off filter (Schott glass) was used to collect light. In all experiments the quoted concentrations are those in the mixing chamber, except where stated. The dead time of the stopped-flow instrument was ∼2 ms; during this initial time, no change in fluorescence can be observed.

Steady-state fluorescence was measured using a Cary Eclipse fluorimeter (Varian) with a xenon light source. Absorbance spectroscopy was performed using a Beckman DU640 spectrophotometer and Cary 50 spectrophotometer (Varian).

##### Kinetic Measurements

All reactions with RecD2 and DNA were done at 20 °C in a buffer containing 20 mm Tris·HCl (pH 7.5), 100 mm sodium chloride, 10 mm magnesium chloride, and 1 mm dithiothreitol. PcrA measurements were performed in a buffer containing 50 mm Tris·HCl (pH 7.5), 150 mm sodium chloride, and 3 mm magnesium chloride. To minimize phosphate contamination, ATPase measurements using MDCC-PBP or rhodamine-PBP were taken in the presence of a P_i_ mop, which comprised of 0.01 unit ml^−1^ bacterial purine nucleoside phosphorylase and 200 μm 7-methylguanosine (22).

Data were fitted to theoretical equations using the stopped-flow software, Kinetic Studio 2, or Grafit (23). Kinetic simulations were performed using Berkeley Madonna (Version 8.3, University of California at Berkeley). All plots of observed rate constants against concentration are the average of three separate experiments.

The fitted equations are as follows: single exponential with amplitude *A_a_* and first order rate constant *k_b_*,

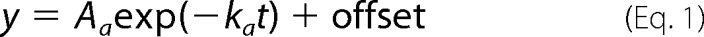
 and double exponential with amplitudes *A_a_* and *A_b_* and first order rate constants *k_a_* and *k_b_*, respectively,

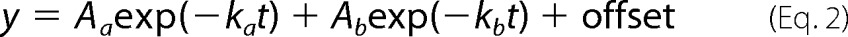


For the linear fits of the change in observed binding rate constants (*k*_obs_) obtained under pseudo-first order conditions with the concentration of one of the species (*C*) in large excess,

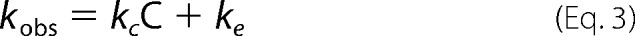
 where *k_c_* is the second order association rate constant, and *k_e_* is the sum of the first order rate constants, controlling breakdown of the bound species.

## RESULTS

### 

#### 

##### Steady-state ATPase Rate Measurements

**FIGURE 2.**
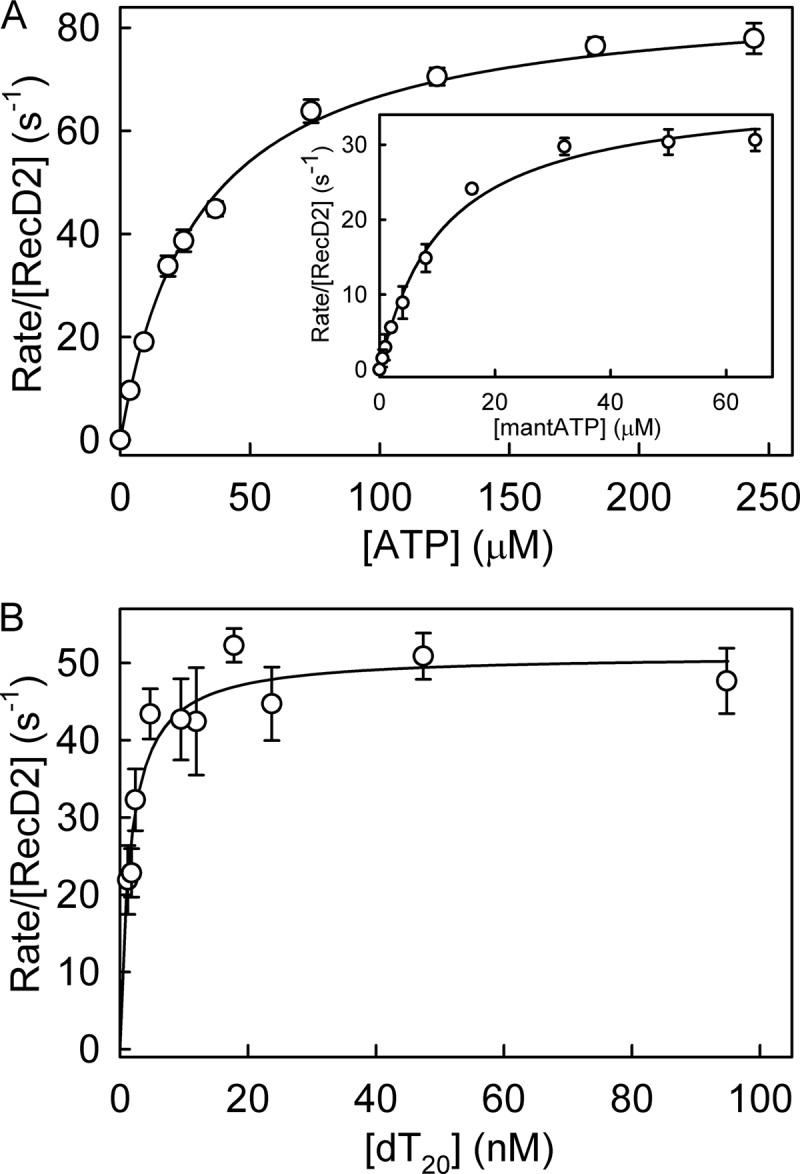
**Steady-state ATPase kinetics for RecD2.** The measurements were carried out at 20 °C with solution conditions as described under “Experimental Procedures” with 1.5 nm RecD2, 500 nm dT_20_, 10 μm MDCC-PBP, and triphosphate nucleotide at the concentrations shown. *A*, shown is are steady-state measurements for ATP. The lines are best fits to the Michaelis-Menten equation to give a *K_m_* of 30.3 ± 1.5 μm and a *k*_cat_ of 86.9 ± 1.9 s^−1^. *Inset*, shown are steady-state measurements for mantATP. The best fit gives a *K_m_* of 9.7 ± 1.3 μm and a *k*_cat_ of 37.3 ± 1.4 s^−1^. *B*, steady-state dependence on ssDNA is shown. Solution conditions are 1.5 nm RecD2, 1 mm mantATP, 10 μm MDCC-PBP, and dT_20_ at the concentrations shown. The lines are best fits to the Michaelis-Menten equation to give a *K_m_* of 1.5 ± 0.5 nm and a *k*_cat_ of 51.1 ± 5.9 s^−1^.

Steady-state parameters were measured to gain an overall assessment of the ATP hydrolysis by RecD2 bound to ssDNA and give an idea of adenosine nucleotide affinities before investigating the individual steps in the ATPase cycle. In all the work described below, dT_20_ was used as the main ssDNA track. The P_i_ biosensor, MDCC-PBP (24), was used to measure steady-state ATP hydrolysis kinetics. In the absence of DNA, the ATPase activity had a *k*_cat_ value of 1.6 s^−1^ (Table 1), relatively high for a helicase alone. Fig. 2*A* shows the rate of ATP hydrolysis as a function of nucleotide concentration in the presence of the ssDNA, giving a *K_m_* value of 30.3 μm and a *k*_cat_ of 86.9 s^−1^. DNA activation of ATPase activity resulted in a 50-fold increase in *k*_cat_ (Table 1). The weak affinity for ATP limits experimentation with this nucleotide due to problems obtaining quantitative complex formation for the key experiments with RecD2 in excess. The fluorescent ATP analog, mantATP, had a *K_m_* of 9.7 μm and *k*_cat_ 37.3 s^−1^ (Fig. 2*A*). The *k*_cat_ value was approximately half that of ATP, and the *K_m_* was 3-fold lower, suggesting significantly tighter binding. Although this *K_m_* value is relatively high compared with other DNA helicases (10, 25), it is possible to form reactive complexes quantitatively with this nucleotide.

**TABLE 1:** **Steady-state ATPase kinetics for RecD2** All measurements were at 20 °C in the presence 10 μm MDCC-PBP, 1.5 nm RecD2, and 500 nm dT_20_ in a buffer described under “Experimental Procedures.”

Nucleotide	*k*_cat_	*K_m_*
	*s*^−*1*^	μ*m*
ATP	86.9 ± 1.5	30.3 ± 1.5
ATP (no DNA)	1.6 ± 0.3	9.4 ± 2.5
MantATP	37.3 ± 1.4	9.7 ± 1.3
MantATP (no DNA)	0.5 ± 0.1	7.8 ± 2.2
Mant-deoxyATP	40.2 ± 2.4	12.1 ± 1.7
MantATPγS	0.3 ± 0.1	8.2 ± 1.9

The affinity of the DNA substrate was determined using the same assay. The ATPase rate as a function of oligonucleotide concentration gave values of *K_m_* for the DNA of 1.5 nm (Fig. 2*B*). Such a low *K_m_* suggested complete binding of RecD2 to the DNA substrate would occur during subsequent measurements. The *k*_cat_ value from this measurement (51.1 s^−1^) was somewhat higher than that from varying ATP values (37.3 s^−1^); the difference may well be due either to getting precise concentrations for the protein or to the P_i_ calibration.

Mant ribonucleotides exist as an ∼1:2 ratio of 2′ and 3′ isomers in relatively rapid equilibrium (14, 26). To determine whether there is a significant difference in ATPase kinetics between the two isomers, steady-state ATPase measurements were carried out with 3′-mant-2′-deoxyATP, for which such isomerization is not possible. The *k*_cat_ was 40.2 s^−1^, and the *K_m_* was 12.1 μm. These parameters are similar to the mixed isomer, suggesting there is not a large difference in activity between the two isomers. As a further test, binding kinetics were measured using 3′-mant-2′-deoxyATP, as described later, showed similar results to the mixed isomers.

To investigate the kinetics of the ATPase cycle, signals are required that report on each individual step. A minimal ATPase cycle is shown in Fig. 1*A*, showing only changes in adenosine nucleotide state, but not addressing the translocation, which is likely to be coupled to particular steps in this cycle. Fluorescent adenine nucleotides, such as mantATP (15), provide signals for binding to and release from proteins as well as potentially for conformation changes elsewhere in the cycle. The use of mantATP enabled a fairly complete set of rate constants to be obtained for the steps in Fig. 1 for a single nucleotide. The rate constants will refer to the numbering scheme in Fig. 1.

##### MantATP Binding to RecD2·ssDNA

**FIGURE 3.**
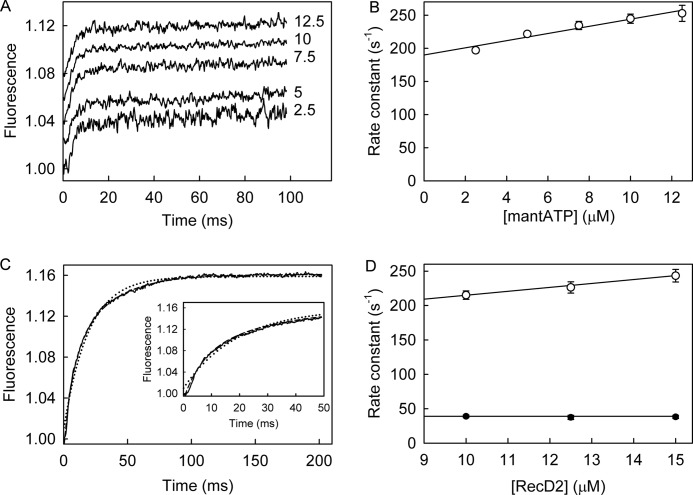
**MantATP binding to RecD2·dT_20_.** MantATP at the micromolar concentrations shown was mixed in the stopped flow apparatus with 0.5 μm RecD2 and 2.5 μm dT_20_ at 20 °C in the buffer described under “Experimental Procedures.” *A*, individual traces (offset from each other) were fitted to single exponentials (Equation 1), and the dependence of the observed rate constants on concentration was then linear-fitted to Equation 3 (*B*). The points shown are averages of at least three measurements. The slope gives *k*_+1_ as 5.4 ± 0.8 μm^−1^s^−1^. The intercept with the ordinate (190 ± 8 s^−1^) represents *k*_−1_ + *k*_+2_ (scheme in Fig. 1*A*). *C*, shown is the time course after mixing 12.5 μm RecD2 and 15 μm dT_20_ with 2.5 μm mantATP. The *inset* shows the initial increase in fluorescence. The complete trace was fitted by a double exponential (Equation 2, *dashed line*), and the single exponential fit is shown for comparison (*dotted line*). The first phase, representing two-thirds of the amplitude, had an observed rate constant of 248 ± 7 s^−1^, and the second phase was 38.4 ± 2.4 s^−1^. *D*, traces were fitted to double exponentials, and the dependence of the observed rate constants on concentration was fitted to Equation 3. The observed rate constant for the initial change in fluorescence is linearly dependent on the RecD2 concentration (*unfilled circles*). After a linear fit to Equation 3, the second order association rate constant was 5.7 ± 0.7 μm^−1^s^−1^, and the intercept was 158 ± 9 s^−1^. The observed rate constants of the second increase in fluorescence (*filled circles*) were independent of RecD2 concentration at 39 s^−1^.

The kinetics for mantATP binding to RecD2·dT_20_ were measured under pseudo-first order conditions with mantATP in large excess over the protein. Using the stopped-flow apparatus, several concentrations of mantATP were rapidly mixed with the RecD2·dT_20_ complex, and fluorescence was followed with time (Fig. 3*A*). The increase in fluorescence was fitted by a single exponential. The observed rate constants were linearly dependent on mantATP concentration (Fig. 3*B*), giving a second order association rate constant of 5.4 μm^−1^s^−1^ from the slope. The intercept with the ordinate (190 s^−1^) is the sum of rate constants controlling breakdown of the bound mantATP, which include both the triphosphate dissociation and hydrolysis.

Extra information may be obtained by mixing excess RecD2·dT_20_ with mantATP and following the fluorescence with time (Fig. 3*C*). This approach is particularly useful because the fluorescent nucleotide can report local rearrangements in the protein during the catalytic cycle due to the much higher signal-to-noise ratio compared with having a large excess of free mant nucleotide (27). The traces showed a biphasic increase in fluorescence. The first rapid phase, which represented two-thirds of the overall change, was followed by a slower increase. The traces were well fitted by a double exponential, and the observed rate constant for the initial phase was linearly dependent of the RecD2 concentration (Fig. 3*D*). Assuming single-step binding, the second order association rate constant was 5.7 μm^−1^s^−1^, and the intercept was 158 s^−1^. The first phase in fluorescence was presumably due to binding mantATP, as both rate constants are very similar to those seen with excess nucleotide. The difference in the dissociation measurements is likely to be a reflection of the accuracy of the intercept determination. The observed rate constant of the second phase, 39 s^−1^, was independent of RecD2 concentration. This second phase is very similar to the steady-state ATPase rate, and therefore, this phase is likely to represent subsequent steps of the mantATP cycle after binding, leading to the formation of mantADP. This then allows the value of *k*_−1A_, the dissociation rate constant, to be determined as 119 s^−1^ (158 − 39) and, hence, the dissociation constant, 1/*K*_1A_ is 21 μm. The RecD2 concentrations used in these measurements were well above the mantADP *K_d_* (5 μm, see below); therefore, most mantADP would remain bound during the time course.

To assess whether the presence of two isomers in mantATP as described above are a factor in the biphasic fluorescence traces described above, 3′-mant-2′-deoxyATP was used in similar measurements with excess RecD2·dT_20_. This single species had a similar biphasic behavior as the mixed isomer (data not shown), consistent with the two-phase change being independent of any preferential binding of either isomer.

**FIGURE 4.**
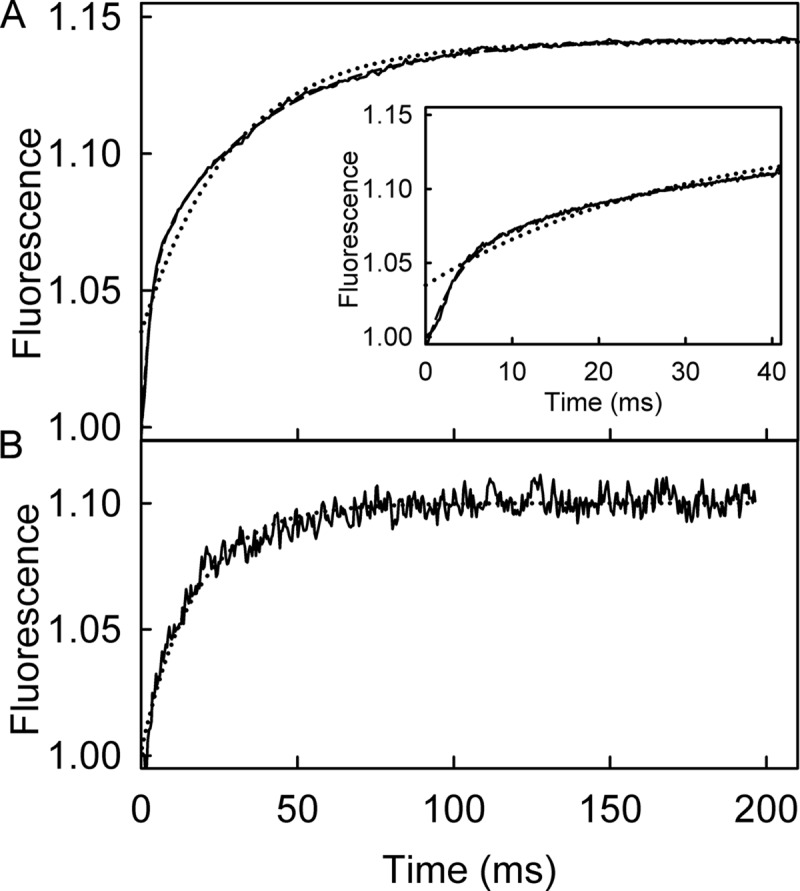
**Mant fluorescent changes upon binding RecD2·dT_20_.** *A*, shown is the time course of mant fluorescence upon mixing 2.5 μm mantATPγS and 12.5 μm RecD2·dT_20_. The *inset* shows the initial increase in fluorescence. The long time traces were fitted to double exponentials (Equation 2, *dashed line*) giving observed rate constants of 256 ± 8 and 27.4 ± 2.3 s^−1^. The single exponential fit is shown for comparison (*dotted line*). *B*, shown is the time course of mant fluorescence upon mixing 2.5 μm mantATP and 12.5 μm RecD2 with 15 μm dT_20_ after a first mixing of 25 μm RecD2 and 5 μm mantATP and aging for 0.1 s. The trace was fitted by a single exponential (Equation 1, *dotted line*) giving a rate constant of 48.2 ± 4.5 s^−1^. If the second mix was with buffer alone (no DNA), there was a small change in fluorescence, which increase linearly over a long time (not shown). This probably represents a basal level of activity.

To probe the basis of the fluorescent changes, the experiments were repeated at a single concentration with the ATP analog, mantATPγS. This analog is hydrolyzed only very slowly by RecD2·dT_20_ (0.3 s^−1^), shown using the steady-state assay described above (Table 1). This was confirmed by HPLC analysis (data not shown). The traces of the single-turnover measurement with mantATPγS in Fig. 4*A* showed a biphasic increase in fluorescence very similar to that with mantATP. The observed rate constants were 256 s^−1^ for the first phase and 27.4 s^−1^ for the second. An equivalent single-turnover P_i_ measurement (as described below for mantATP) showed no P_i_ formation over the time period of the mant measurement, confirming that this nucleotide was essentially not hydrolyzed on this time scale. The two fluorescence phases observed with mantATPγS are very likely to be related to binding and a subsequent conformation change to the mantATPγS complex but cannot be due to cleavage.

Because the second phase with mantATP is similar to that with mantATPγS, it is assumed that the mantATP complex undergoes a pre-cleavage conformation change, as shown in the scheme of Fig. 1*B*, to give rise to this second phase. To investigate this further, a double-mix, stopped-flow experiment was performed with mantATP to see if the biphasic increase in fluorescence could be separated into the individual processes. An excess of RecD2 was first mixed with mantATP, and this mixture was aged for 0.1 s to allow binding but not hydrolysis, which is relatively slow in the absence of DNA (Table 1). This solution was then mixed with excess dT_20_. The fluorescent trace (Fig. 4*B*) showed a single exponential increase in mant fluorescence with a rate constant of 48.2 s^−1^. This rate constant was similar to that of the second, slower phase, measured above, consistent with this phase being due to a process in the DNA-activated ATPase pathway, subsequent to initial binding.

##### Hydrolysis Step and P_i_ Release

**FIGURE 5.**
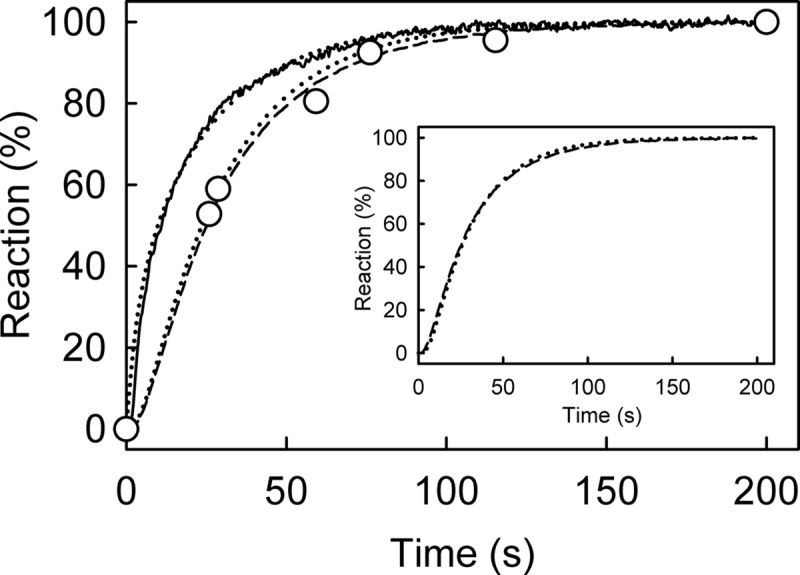
**Kinetic measurement of mantATP with excess of RecD2·dT_20_: binding, hydrolysis, and P_i_ release.** Time course of mant fluorescence (*solid line*), mantADP formation (*circles*), and P_i_ release (*dashed line*) is shown. All measurements were at 2.5 μm mantATP, 12.5 μm RecD2, 15 μm dT_20_, and 10 μm MDCC-PBP (for P_i_ measurement) and were carried out under the conditions of Fig. 3 as described under “Experimental Procedures.” The time courses were simulated (*dotted lines*), based upon a global model for a single turnover of mantATP based on the scheme in Fig. 1*B*, as described under “Results.” The simulated mant fluorescence and cleavage time course are in the main panel, and the P_i_ simulation with the experimental data is in the *inset*. This gave an observed first order rate constant for mantATP binding ([RecD2] × *k*_+1_*_a_*) at 200 s^−1^ followed by the conformation change (*k*_+1_*_b_*) at 54 s^−1^, hydrolysis (*k*_+2_) at >300 s^−1^; P_i_ release (*k*_+3_) was fast (>300 s^−1^).

The single-turnover hydrolytic cleavage was compared with the mantATP fluorescence measurements under similar conditions of excess RecD2·dT_20_ over mantATP, as described above. In this way binding, hydrolytic cleavage, and product release can be directly related to each other. Quenched-flow measurements allowed the formation of diphosphate to be monitored with high time resolution (Fig. 5).

Using the phosphate biosensor, MDCC-PBP, the kinetics of P_i_ release were also measured under similar conditions (Fig. 5). These are similar to the rate constant for cleavage, so P_i_ release follows rapidly after this.

##### Simulation of the ATPase Cycle

A simple model for the ATPase mechanism based on the scheme in Fig. 1*B* was used to simulate the data in Fig. 5. P_i_ release was assumed to be irreversible in the model because its measurement was in the presence of MDCC-PBP, which would sequester free P_i_ and so limit any possibility of rebinding. The slow step in the fluorescence traces was assumed to be a conformation change within the triphosphate complex (*step 1B* in Fig. 1*B*), and the rationale for this will be developed under “Discussion.” A difference in fluorescence between protein-bound mantATP (before the conformation change) and mantADP was included in the modeling with step 1A representing two-thirds of the overall increase, with a subsequent one-third increase going to bound mantADP, as shown experimentally below. The best-fit simulation is shown in Fig. 5. This gave mantATP binding at 200 s^−1^ (*k*_+1_*_a_* × [RecD2]; Fig. 1*B*) followed by a conformation change 54 s^−1^ (*k*_+1_*_b_*) then hydrolytic cleavage (*k*_+2_) and P_i_ release (*k*_+3_), both significantly faster, assumed to be >300 s^−1^. MantADP release was not included in the simulation as under the conditions of the measurements, most mantADP remains bound. This suggests that the cleavage and subsequent P_i_ release kinetics are controlled by the slow conformation change (step 1B).

##### MantADP Association and Dissociation Kinetics

**FIGURE 6.**
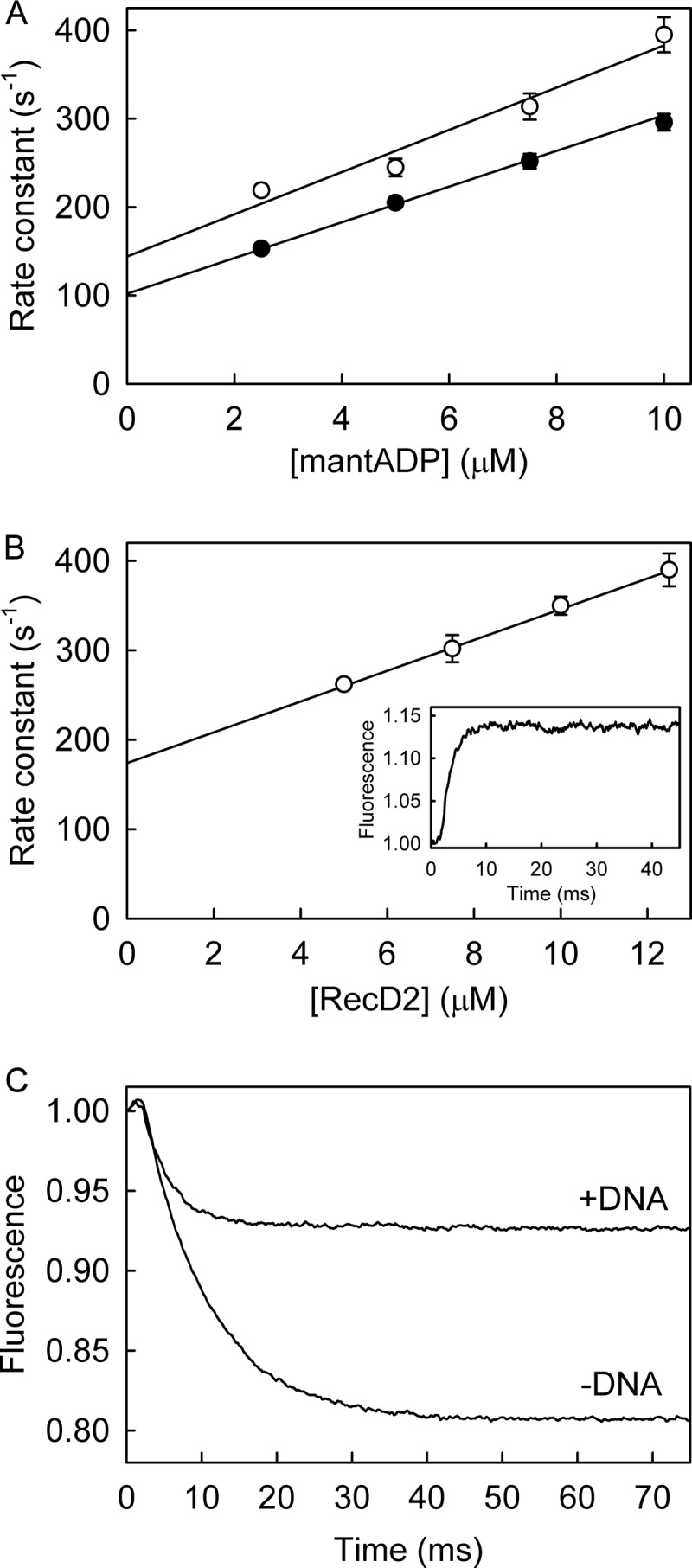
**MantADP binding kinetics to RecD2·dT_20_.** *A*, mantADP at various concentrations was mixed in the stopped flow apparatus with 0.5 μm RecD2 with (*open circles*) or without (*filled circles*) 2.5 μm dT_20_ under the conditions of Fig. 3. Traces were fitted by single exponentials (Equation 1). The rate constants are shown as a function of concentration and the best linear fit (Equation 3) and give an association rate constant of 23.9 ± 3.6 μm^−1^ s^−1^ and a dissociation rate constant of 144 ± 25 s^−1^. *B*, RecD2 at various concentrations was mixed against 2.5 μm mantATP in the presence of 15 μm dT_20_. The traces were fitted by single exponentials, and the resulting observed rate constants are plotted against concentration. A linear fit (Equation 3) gives an association rate constant with dT_20_ bound of 17.2 ± 1.3 μm^−1^ s^−1^ from the slope and a dissociation rate constant of 174 ± 5 s^−1^ from the intercept. In the absence of DNA, the fit gives an association rate constant of 20.2 ± 1.2 μm^−1^ s^−1^ and dissociation rate constant of 102 ± 9 s^−1^. *Inset*, shown is the time course of an excess of 12.5 μm RecD2 (15 μm dT_20_) binding 2.5 μm mantATP. *C*, MantADP release from RecD2·dT_20_ is shown. 12.5 μm RecD2 was premixed with 2.5 μm mantADP in the presence or absence of 15 μm dT_20_ before being rapidly mixed against 200 μm ADP. The time course, after the apparent lag due to the dead time of the stopped flow instrument (∼2 ms), was fitted to a single exponential giving a dissociation rate constant of 240 ± 3 s^−1^.

MantADP binding to the RecD2·dT_20_ complex was measured under pseudo-first order conditions, excess mantADP over protein, in the stopped-flow apparatus. Fig. 6*A* shows a linear dependence between the observed rate constants and mantADP concentration. The slope gave a second order rate constant (*k*_-4_ in the scheme of Fig. 1) of 23.9 μm^−1^ s^−1^, and a dissociation rate constant (*k*_+4_) of 144 s^−1^ from the intercept, giving *K*_4_ as 5.8 μm.

Binding kinetics were also measured with excess RecD2·dT_20_ over mantADP (Fig. 6*B*). The traces were well fitted by single exponentials, giving an association rate constant of 17.2 μm^−1^ s^−1^ and a dissociation rate constant of 174 s^−1^, in the same range as those measured with an excess of mantADP.

The measurements were under the same conditions as the mantATP binding, hydrolysis, and P_i_ release measurements (Figs. 3*C* and 5), namely with excess RecD, so that essentially all nucleotides bound. Given that mantADP and mantATP free in solution have identical fluorescence intensities (14), this allowed a direct comparison of the fluorescence levels between bound mantATP including its subsequent conformation change and cleavage (Fig. 3*C*) and bound mantADP (Fig. 6*B*). The overall fluorescence change was similar for the two measurements, suggesting that the end point in each case is bound mantADP with a similar conformation. Thus there is an extra 50% increase in fluorescence ongoing from initially bound mantATP (after step 1A) to bound mantADP (Fig. 1*B*), as used in the simulation above.

The relatively tight binding of mantADP allowed direct measurement of dissociation kinetics from a preformed complex. Excess RecD2·dT_20_ was premixed with mantADP and then mixed rapidly in a stopped-flow apparatus with excess, unlabeled ADP to act as a trap for RecD2 after its dissociation from mantADP. The mant fluorescence decreased with time, and the traces were fitted by a single exponential (Fig. 6*C*). The observed rate constant, equivalent to *k*_+4_ (Fig. 1), 240 s^−1^, was independent of ADP in the range of 200–800 μm. This measurement gave a somewhat faster rate constant than those measured from the intercept in the binding measurements (174 s^−1^).

##### Measurements in the Absence of DNA

To determine which parts of the ATPase cycle are modulated by the interaction with DNA, the kinetics of specific steps were measured in the absence of DNA. Using ATP as substrate in steady-state measurements, *k*_cat_ was 50-fold less than in the presence of ssDNA (Table 1). Furthermore, the double-mix experiments, described above, showed that binding DNA induces the second mant fluorescence phase, suggesting that interaction of the helicase with ATP and with DNA modulate each other.

**FIGURE 7.**
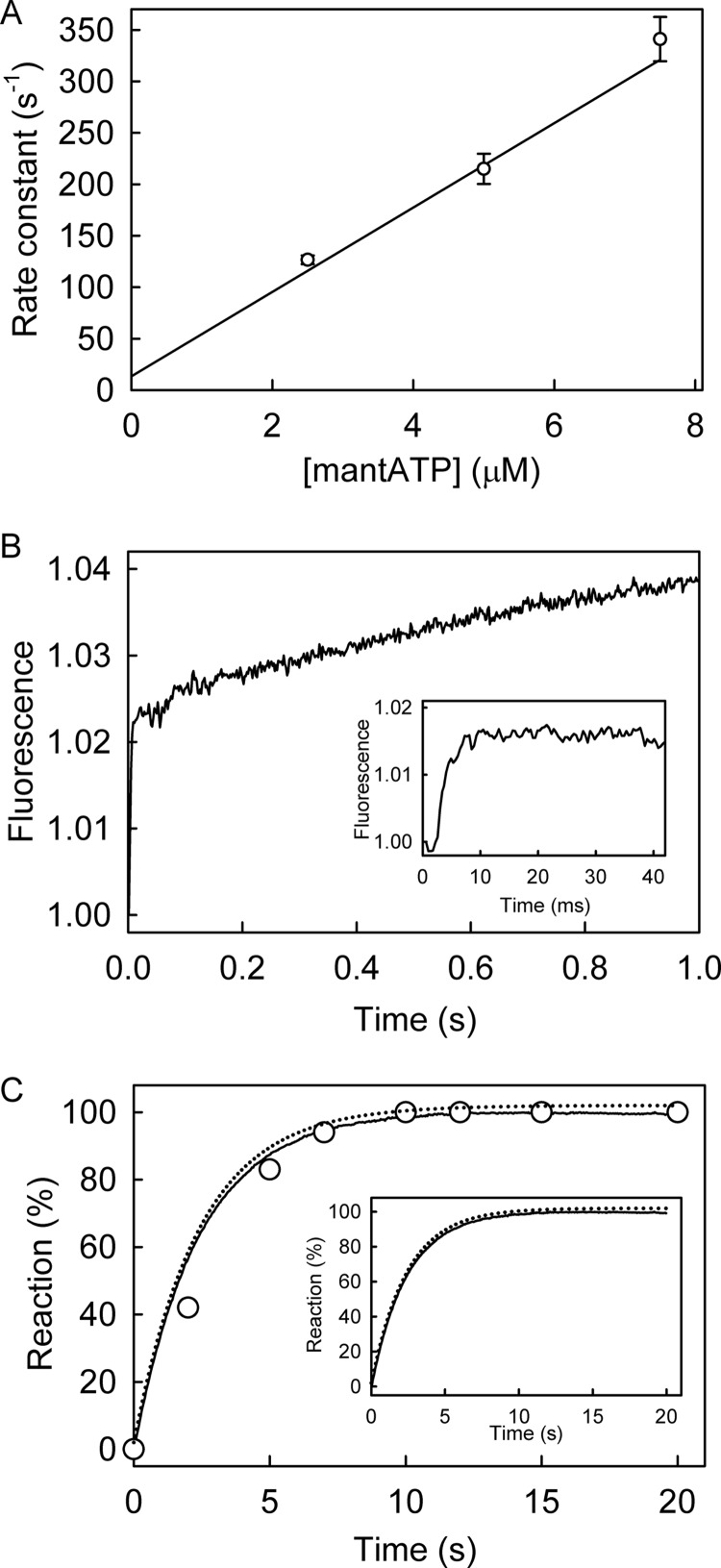
**MantATP measurements in the absence of DNA.** *A*, MantATP at the micromolar concentrations shown was mixed in the stopped flow apparatus with 0.5 μm RecD2 under the conditions of Fig. 3. Traces were fitted by single exponentials (Equation 1), and the dependence of the observed rate constants on concentration was then linear fitted to Equation 3. The fit gives an association rate constant of 42.8 ± 4.4 μm^−1^s^−1^. The intercept gives the dissociation rate constant of 13.4 ± 5.8 s^−1^. *B*, the time course of 12.5 μm RecD2 binding 2.5 μm mantATP is shown. The *inset* shows the initial increase in fluorescence. Fitting the first phases, after the apparent lag due to the dead time of the stopped flow instrument (∼2 ms), to a single exponentials gave an observed rate constant of 296 ± 22 s^−1^. The slow second phase is probably the slow hydrolysis. *C*, shown is the time course of mantADP formation (*circles*) and P_i_ release (*solid line*), measured as described under “Experimental Procedures.” The simulation from Fig. 5 was applied to fit the acid quench (*dotted line in the main panel*) and P_i_ release data (*dotted line in the inset* together with the data). Both simulated lines are offset by 2% for clarity This gave the observed first order rate constant for mantATP binding at 250 s^−1^ followed by a conformation change 0.5 s^−1^ followed by fast hydrolytic cleavage and P_i_ release.

To measure association kinetics of RecD2 and mantATP, RecD2 was mixed with excess mantATP under pseudo-first order conditions in the stopped-flow apparatus, and the fluorescence was followed with time. After fitting by single exponentials, there was a linear relationship between the observed rate constant and nucleotide concentration (Fig. 7*A*). Interpreting the increase in terms of single-step binding gave a second order association rate constant of 42.8 μm^−1^s^−1^ and an intercept of 13.4 s^−1^. The association rate constant was significantly faster than that measured with dT_20_, and the intercept was much lower, only in part explained by the slower hydrolysis. Subtracting the hydrolysis rate constant (0.5 s^−1^) from the intercept gives the dissociation rate constant as 12.9 s^−1^, so that the dissociation constant was 0.3 μm, 2 orders of magnitude tighter than in the presence of ssDNA.

As done for the RecD2·dT_20_, measurements were also taken with an excess of RecD2 over mantATP (Fig. 7*B*). A rapid increase in fluorescence was observed followed by a small gradual increase. Fitting the first phases to a single exponentials gave an observed rate constant of 296 s^−1^. Although slower than the rate constant obtained above, this agrees with the process being very fast. The subsequent slow phase probably represents hydrolysis, consistent with the slow steady-state rate in the absence of DNA.

Under the same conditions of excess RecD2 over mantATP, as above, cleavage and P_i_ release were measured (Fig. 7*C*). The hydrolysis time course, measured by manual quench, was similar to that obtained for P_i_ release. The model used to fit data in Fig. 5 was applied to fit the acid quench and P_i_ release data. The simulation used the following parameters: mantATP binding at 250 s^−1^ followed by a conformation change 0.5 s^−1^, then hydrolytic cleavage and P_i_ release, both fast, >10-fold faster than the conformation change. Note that the data cannot give accurate measurements of these last two rate constants, and they are merely assumed to be fast. The conformation change controlling hydrolysis, was ∼100-fold slower than in the presence of DNA. The time-course of mantADP and P_i_ release were the same as the second phase of the mant fluorescence. This shows an important correlation between the nucleotide fluorescence signal and product formation.

The association kinetics of mantADP to RecD2 were measured by rapidly mixing excess mantADP with RecD2 in the stopped-flow apparatus. A linear dependence between the observed rate and mantADP concentration was seen (Fig. 6*A*). This gave an association rate constant of 20.2 μm^−1^ s^−1^ and a dissociation rate constant of 102 s^−1^. The rate constants suggest a dissociation constant of 5 μm, similar to that seen in the presence of ssDNA but 10-fold weaker than mantATP in the absence of DNA.

The mantADP dissociation experiments were repeated in the absence of DNA (Fig. 6*C*). An excess of RecD2 was prebound with mantADP before rapid mixing against an excess of ADP. The single exponential decrease in fluorescence had a rate constant of 115 s^−1^, similar to the value obtained from the binding experiments above.

##### Measurements with Unlabeled Nucleotides

For comparison with the mantATP kinetics, measurements were made where possible with the natural substrate. There is no intrinsic change in protein tryptophan fluorescence change that could be used to measure binding kinetics of ATP or ADP directly.

**FIGURE 8.**
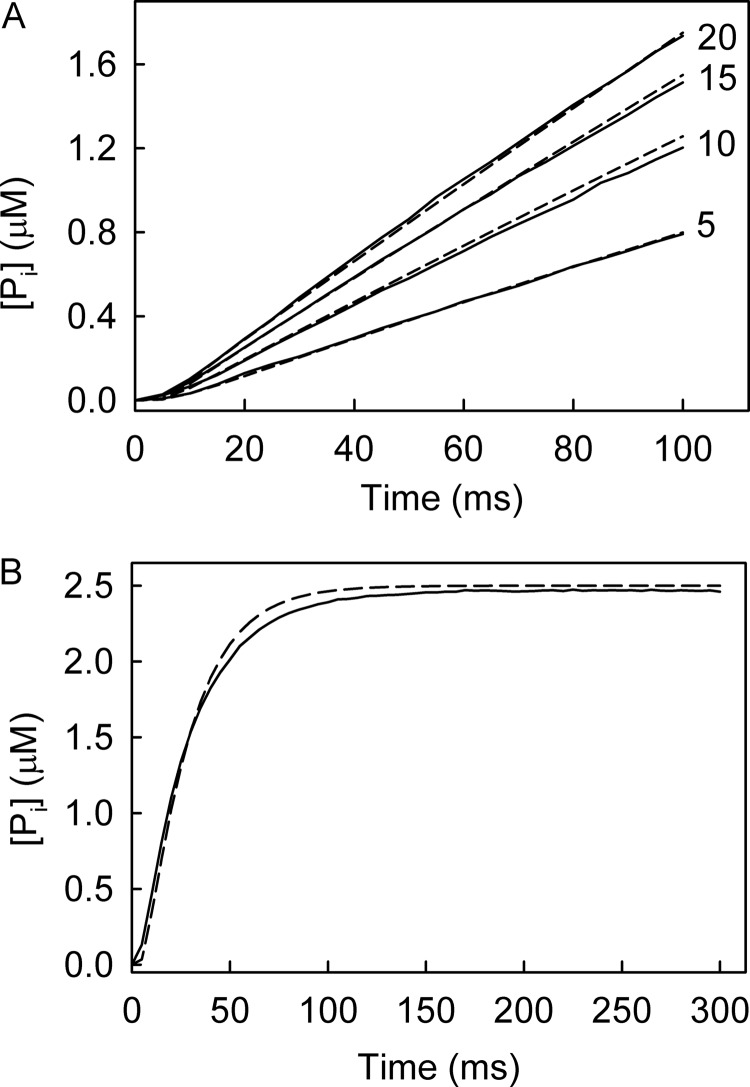
**Measurements with unmodified nucleotides.** *A*, shown are phosphate release measurements at different ATP concentrations. 0.5 μm RecD2 with 2.5 μm dT_20_ was mixed in the stopped-flow apparatus with ATP at the micromolar concentrations shown in the presence of 10 μm MDCC-PBP. The simulated time courses are *dashed lines*. This fitting gave a rate constant for ATP binding as 8 μm^−1^s^−1^, dissociation of ATP as 40 s^−1^, conformation change as 85 s^−1^; hydrolysis and P_i_ release was fast >250 s^−1^, and the ADP release was >300 s^−1^ with ADP association as 8 μm^−1^s^−1^. *B*, phosphate release measurements from a single turnover are shown. 12.5 μm RecD2 (15 μm dT_20_) was mixed with 2.5 μm ATP in the presence of 10 μm MDCC-PBP under the conditions of Fig. 5. The simulated trace is the *dashed line* based upon the same parameters used in *A*.

The rate of P_i_ release was measured by mixing RecD2·dT_20_ with excess ATP using MDCC-PBP in the stopped-flow apparatus (Fig. 8*A*). The traces showed a slight lag followed by a fairly linear P_i_ release, which increased in slope with ATP concentration. The model of the ATPase cycle (Fig. 1*B*) was then used to simulate these data to obtain individual rate constants (Fig. 8*A*). This gave rate constants for ATP binding as 8 μm^−1^ s^−1^, dissociation of ATP as 40 s^−1^, conformation change as 85 s^−1^, hydrolysis and P_i_ release, fast, >250 s^−1^, and the ADP release was >300 s^−1^ with ADP association as 8 μm^−1^s^−1^.

The P_i_ release assay was repeated under conditions of excess RecD2·dT_20_ mixed with ATP in the presence of the phosphate biosensor (Fig. 8*B*). The trace showed a lag phase then an exponential increase in fluorescence. This reaction represents a single ATP turnover, therefore, assuming ADP release is the final step in the mechanism; the ADP affinity does not affect the simulation. The data were fit well by the same parameters as those in Fig. 8*A*, suggesting that the model rate constants are an accurate representation of the ATP cycle. However, it should be noted that the binding rate constant is mainly determined by the size of the short lag, and this feature is difficult to fit.

##### The Role of Magnesium in DNA Activation of the ATPase Cycle

In PcrA helicase, Mg^2+^ is coordinated to residue Lys-37 in the crystal structural (28). This amino acid residue moves out of the Mg^2+^ binding site upon DNA binding, which allows Mg^2+^ to bind there and subsequent catalysis to occur. To investigate the presence of a similar effect in RecD2, measurements were performed in the absence of magnesium and with (K336A)RecD2. This lysine is the equivalent of Lys-37 in PcrA.

**FIGURE 9.**
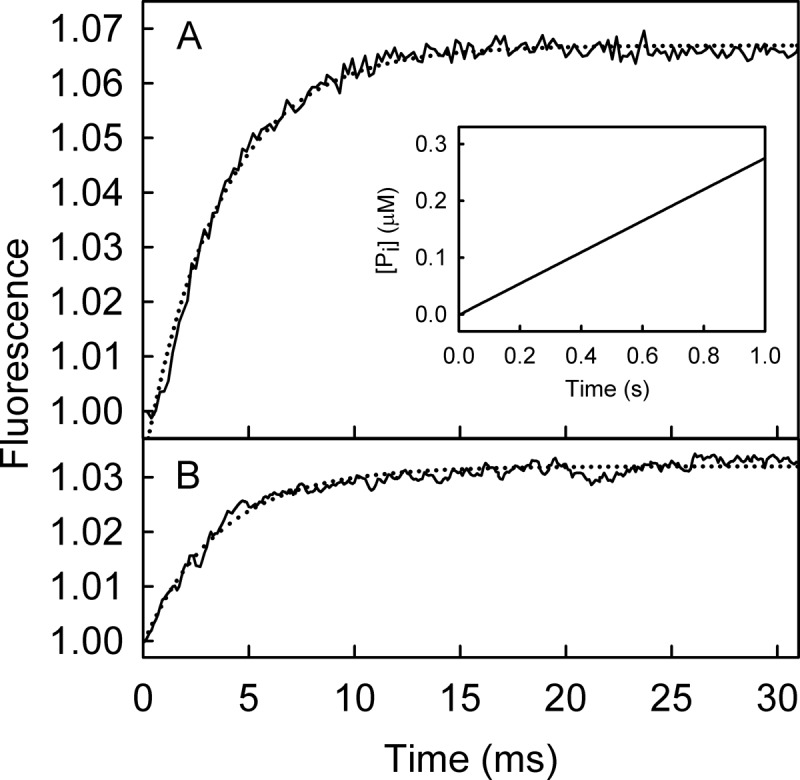
**MantATP binding to RecD2·dT_20_ in the absence of Mg^2+^.** *A*, shown is the time course of 12.5 μm RecD2 (15 μm dT_20_) binding 2.5 μm mantATP under the conditions of Fig. 5, with the exception that Mg^2+^ was not added and 5 mm EDTA was included in the buffer. The traces were fitted by a single exponential (*dotted line*) giving a rate constant of 262 ± 4 s^−1^. *Inset*, shown is the time course of P_i_ release from the same mixture in the presence of MDCC-PBP. A linear fit gives a rate constant (rate/[P_i_]) 0.03 s^−1^. *B*, shown is the time course of 12.5 μm (K336A)RecD2 (15 μm dT_20_) binding 2.5 μm mantATP under the conditions of Fig. 5. The time course after the apparent lag due to the dead time of the stopped flow instrument (∼2 ms) was fitted by a single exponential (*dotted line*) with rate constant of 251 ± 6 s^−1^.

MantATP binding was measured in the absence of magnesium using a buffer without Mg^2+^ but with EDTA present to sequester any there fortuitously (Fig. 9*A*). Experiments with excess protein were performed, equivalent to Fig. 3*C*. The fluorescence traces were fitted by a single exponential giving an observed rate constant of 262 s^−1^. There was no observable second phase, indicating that the pre-cleavage conformation change may not occur. P_i_ measurements to measure cleavage were performed in the presence of MDCC-PBP and gave an observed rate constant of <0.03 s^−1^ (Fig. 9*A*, *inset*). This shows that the hydrolysis step is greatly affected by Mg^2+^, as would be expected.

Using (K336A)RecD2, the ATPase measurements were repeated under the conditions of Fig. 5; that is, with Mg^2+^ present (Fig. 9*B*). The fluorescence traces for mantATP binding were fitted to a single exponential and gave an observed rate constant of 251 s^−1^. Again, there was no observable second phase in mant fluorescence and essentially no hydrolysis. This highlights that Mg^2+^ access and coordination is one of the steps that leads to DNA activation of RecD2 helicase.

##### Pre-hydrolysis Conformation Change in PcrA Helicase?

The second fluorescent phase, observed when binding mantATP with excess RecD2 (Fig. 5), was correlated with a pre-cleavage conformation change. Such a step was not observed with PcrA using mant nucleotides (10). This may be due to a fundamental difference between these helicases or due to differences in experimental conditions. It is possible that the process was observed with RecD2 because of the relatively tight diphosphate binding combined with the experimental conditions, leading to most diphosphate remaining bound during the single turnover binding and hydrolysis measurements (Fig. 5). In contrast, PcrA has relatively weak ADP and mantADP affinity, and the equivalent measurements reported for PcrA included at least partial dissociation of mantADP (10). Any fluorescence change due to a pre-cleavage conformation change may have been hidden between the increase in fluorescence on binding mantATP by the decrease on mantADP release.

**FIGURE 10.**
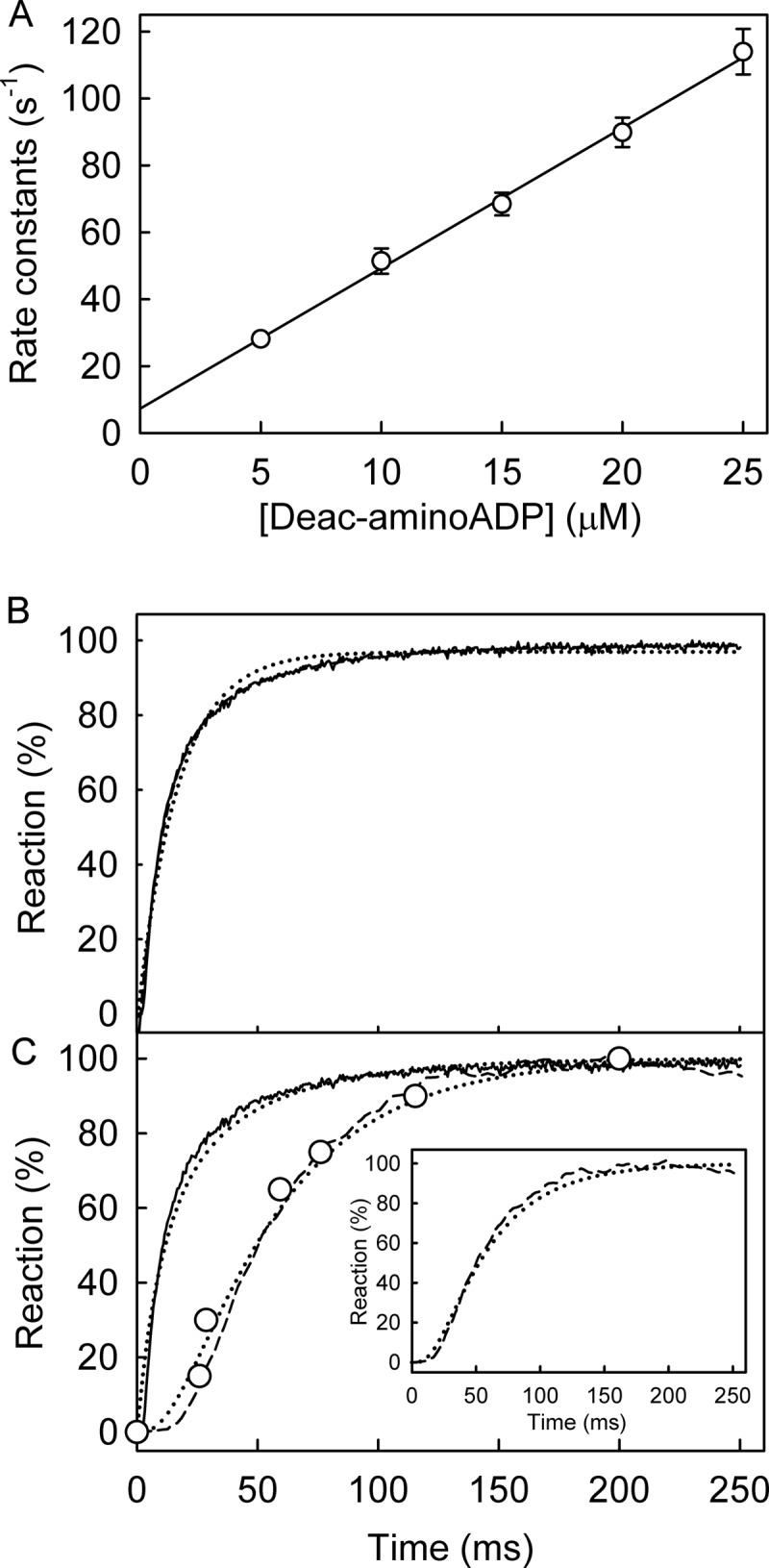
**PcrA helicase ATPase kinetics with deac-aminoATP: binding, hydrolysis, and P_i_ release.** *A*, shown is PcrA·dT_20_ binding deac-aminoADP. Deac-aminoADP at various concentrations was mixed in the stopped flow apparatus with 0.5 μm PcrA and 2.5 μm dT_20_. Traces were fitted by single exponentials. The observed rate constants are shown as a function of concentration, and the best linear fit (Equation 3) gives an association rate constant of 4.2 ± 0.3 μm^−1^ s^−1^ and a dissociation rate constant of 7.3 ± 2.2 s^−1^, resulting in a dissociation constant of 1.7 μm. *B*, shown is deac-aminoATP binding with excess RecD2. The concentrations were 2 μm deac-aminoATP, 8 μm PcrA, 10 μm dT_20_. The time course of fluorescence (*solid line*) was fitted by a double exponential. The initial increase in fluorescence had a rate constant of 130 ± 3 s^−1^, and this was followed by a slower change in fluorescence with a rate constant of 26.2 ± 0.9 s^−1^. *C*, shown is deac-aminoATP cleavage and P_i_ release. The concentrations were as for *panel B*. The binding traces were repeated from *panel B*. Deac-aminoADP formation (*circles*) was measured using quench flow and HPLC. P_i_ release (*dashed line*) was measured in the presence of 10 μm rhodamine-PBP. The simulation in Fig. 5 was applied to fit the dataset. The simulated binding and cleavage are the *dashed line* in the *main panel*. The simulated P_i_ release is in the *inset*. This gave the observed first order rate constant for deac-aminoATP binding (equivalent to [PcrA] × *k*_+1_*_a_*) at 100 s^−1^ followed by a conformation change (equivalent to *k*_+1_*_b_*) at 29 s^−1^, hydrolytic cleavage at >100 s^−1^ and P_i_ release at >300 s^−1^.

To test this hypothesis, a single turnover measurement, equivalent to Fig. 5, was done with PcrA helicase but using the fluorescent ATP analog, deac-aminoATP (16). Deac-aminoADP has a significantly tighter interaction with PcrA with a *K_d_* of 1.7 μm (Fig. 10*A*), so almost all the diphosphate should remain bound during the single turnover experiment. After mixing excess PcrA·dT_20_ with deac-aminoATP, there was a biphasic increase in fluorescence (Fig. 10*B*). The initial increase in fluorescence, which accounted for 75% of the overall fluorescence change, had a rate constant of 130 s^−1^. This was followed by a slower change in fluorescence with a rate constant of 26 s^−1^. The first phase in fluorescence is presumably binding, whereas the second change could be due to a pre-cleavage conformation change. This was supported by measurements of hydrolysis by quench-flow and P_i_ release using a phosphate biosensor under the same conditions (Fig. 10*B*). To show the similarity in the mechanism between RecD2 and PcrA, the same model, used to fit data in Fig. 5, was applied to fit this dataset (Fig. 10*C*). This gave deac-aminoATP binding with a first order rate constant (equivalent to [PcrA] × *k*_+1_*_a_*; Fig. 1*B*) of 100 s^−1^ followed by a conformation change binding (equivalent to *k*_+1_*_b_*) of 29 s^−1^, hydrolytic cleavage at >100 s^−1^, and P_i_ release at >300 s^−1^.

## DISCUSSION

### 

#### 

##### ATPase Cycle

Individual rate constants in the ATPase cycle were measured to determine which steps were modulated by ssDNA and which nucleotide-bound intermediates are the main ones present during translocation. Mant-nucleotides were used for this together with ssDNA, so there were fluorescent signals for different steps of the cycle. The main set of rate constants is summarized in Table 2. An assessment of some rate constants for ATP was made by measuring the kinetics of P_i_ release at various concentrations of ATP. The traces were fitted to the same model used for mantATP (Fig. 1*B*). Overall, this showed that there are no large differences from the mechanism proposed with mantATP.

**TABLE 2:** **Summary of individual rate constants for the hydrolysis cycle of mantATP in the presence of DNA** The parameters are defined from schemes A and B in Fig. 1 and were determined at 20 °C.

Parameter	Value	S.E.
*k*_+1_*_a_*	5.4 μm^−1^s^−1^	0.8 μm^−1^s^−1^
*k*_−1_*_a_*	119 s^−1^	9 s^−1^
*k*_+1_*_b_*	34.5 s^−1^	1.8 s^−1^
*k*_+2_	>300 s^−1^	
*k*_+3_	>300 s^−1^	
*k*_+4_	240 s^−1^	3 s^−1^
*k*_−4_	23.9 μm^−1^ s^−1^	3.6 μm^−1^s^−1^

Binding kinetics for mantATP were measured with excess RecD2·DNA. This enabled conditions to be obtained in which all nucleotide binds as triphosphate and largely remains bound as diphosphate. The fluorescent signal could then be used to monitor processes within the ATPase cycle as well as triphosphate binding and diphosphate release. On binding mantATP, two fluorescent phases were observed (Fig. 5*A*). The first represented binding *per se* (*step 1A* of Fig. 1*B*), and the second had kinetics corresponding to the hydrolytic cleavage from the triphosphate- to diphosphate-bound complex, as shown by quench-flow. The evidence that this second phase is a kinetically distinct conformation change between triphosphate binding and cleavage (step 1B) is mainly based on the similar two-phase fluorescence transient when mantATPγS was used (Fig. 4*A*) even though there was no hydrolysis on this time scale. This suggests that with mantATP, the pre-cleavage conformation change was followed by rapid cleavage (step 2) and P_i_ release (step 3).

##### Implications of a Pre-cleavage Conformation Change

The rate-limiting step in the whole cycle is, therefore, the postulated conformation change immediately before cleavage (step 1B). From structural evidence, it was predicted that conformation changes occur upon ATP binding and hydrolysis, so that the ATPase cycle shuttles the structure between two main conformations to produce DNA movement (7). Our data refines this mechanism by providing evidence for a kinetically distinct step before the hydrolytic cleavage, which potentially represents the conformational change required for that structural model. The fluorescence data suggest that this conformation may then be maintained through to bound-ADP, but then the “reset” conformational change occurs either on ADP release or ATP binding.

To obtain more information about this conformation change, single-turnover kinetics were measured in the absence of Mg^2+^. With these conditions, hydrolysis is very slow (Fig. 9*A*). The mant fluorescence transient showed only binding with a rate similar to that in the presence of Mg^2+^. This suggested that the binding was not affected by the triphosphate being bound by Mg^2+^. (K336A)RecD2 was also used to probe the possible effects of Mg^2+^. The equivalent residue in PcrA (Lys-37) moves out of the Mg^2+^ binding site upon DNA binding, allowing Mg^2+^ to bind in that site, and Lys-37 coordinates with the metal (28). ATPase measurements with (K37A)PcrA showed dramatically reduced activity (28). Using (K336A)RecD2 in the single turnover fluorescence measurement, only the initial phase (binding) is present with essentially no hydrolysis. Therefore, it is possible that this residue in RecD2 has the same function as in PcrA. The absence of the second phase is consistent with the conformation change requiring Mg^2+^ and so may involve a repositioning, or coordination, of the metal ion ready for hydrolysis. This mechanism implies that a rearrangement in the Mg^2+^ binding site occurs with every catalytic cycle and is required for ATP cleavage. Such a change would be in tandem with the binding and release of the RecA-like domains as the helicase required for physical movement along the ssDNA.

The conformation change, observed with ssDNA, is directly affected by DNA; the rate constant was reduced 50-fold without DNA. Furthermore, a double-mix stopped-flow experiment, which premixed RecD2 with mantATP, showed that subsequent addition of DNA generates fluorescence, equivalent to the second phase in the transient of Fig. 5*A*. This supports the idea that the conformation change represents the key step in producing cleavage and, by relating to the structural changes (7), seems likely to be key for translocation.

##### A Generic ATPase Mechanism for SF1 Helicases?

How does this mechanism of RecD2, a 5′-3′ SF1 helicase, relate to others in the same family, particularly PcrA, a well studied SF1 helicase with opposite polarity? Some similarities are rapid triphosphate binding and P_i_ and ADP release, albeit that with PcrA a slower process was also observed during mantADP release. No evidence was found for a pre-cleavage conformation change with PcrA, and it was concluded that the key structural change could be during the rate-limiting cleavage step itself (10), although that did not preclude a change that was kinetically indistinguishable. The data with RecD2 suggested the possibility that the reason no separate conformation change was seen for PcrA was not its absence but, rather, the experimental conditions. Under those conditions, significant mantADP would dissociate rapidly from the PcrA·DNA complex after hydrolysis and fluorescence change of that dissociation might mask any small change due to a conformation change.

To test this idea and help relate the mechanism of the two helicases, the single turnover ATPase measurement for PcrA was repeated with a fluorescent ATP analog whose diphosphate has much tighter binding to the protein and so would remain bound under the experimental conditions. In this case (Fig. 10*B*), a second fluorescence phase was observed with PcrA, equivalent to that with RecD2. In other words, this shows that PcrA may have a similar mechanism at this stage of the cycle. The importance of this pre-cleavage conformation change is highlighted by the fact that it is this step that is rate-limiting and, therefore, controls progression of the whole cycle. This suggests that, despite the different polarities of movement, RecD2 and PcrA have similar ATPase mechanisms.

In the case of PcrA, it was proposed that ATP hydrolysis requires obligatory directed movement on DNA, either ssDNA translocation or dsDNA unwinding (11). A pre-cleavage conformation change in effect provides a trigger both for movement by one base and for ATP hydrolysis. The PcrA motor unwinds DNA with a passive mechanism whereby there is a risk of futile ATP hydrolysis. However, PcrA tightly couples ATP hydrolysis with DNA translocations and unwinding. Similarly, RecD2 tightly couples ATP hydrolysis, at least with ssDNA translocation (7). Therefore, the mechanism presented here demonstrates how this can be achieved to prevent futile cycles of hydrolysis.

Other helicase studies have shown hydrolysis or product release to be the rate-limiting step in the cycle (25, 29–33). It is possible that the RecD2 mechanism represents a general mechanism for many monomeric helicases of SF1; a pre-cleavage conformation change of the triphosphate state is rate-limiting. With respect to SF2, there is evidence in a number of helicases for a pre-cleavage conformation change, but product release may be rate-limiting. With RecG, there is slow ADP release (25), and with DEAD-Box helicases P_i_ release is generally at least partly rate-limiting (29, 30, 32, 33).

In summary, each step of the ATPase cycle for RecD2 was measured. It is proposed that a pre-cleavage conformation change is the rate-limiting step in the cycle. This step is regulated by DNA, which controls Mg^2+^ access and coordination. There is evidence for a similar mechanism with the SF1A helicase, PcrA, so this could be a common mechanism for SF1 members. There are no significant differences in mechanism depending on the translocation direction of the motor.

## Footnotes

3 The abbreviations used are:

SF: helicase superfamily
MDCC-PBP: phosphate-binding protein (PBP) (A197C) from *E. coli*, labeled with MDCC (*N*-[2-(1-maleimidyl)ethyl]-7-diethylaminocoumarin-3-carboxamide)
rhodamine-PBP: phosphate-binding protein (A17C/A197C) from *E. coli*, labeled with 6-IATR (6-iodoacetamidotetramethylrhodamine)
ADPNP: βγ-imido-adenosine-5′-triphosphate
mant: *N*-methylanthraniloyl
mantATP: 2′(3′)-*O*-(*N*-methylanthraniloyl)ATP
mant-deoxyATP: 3′-*O*-(*N*-methylanthraniloyl)-2′-deoxyATP
deac-aminoATP: 3′-(7-diethylaminocoumarin-3-carbonylamino)-3′-deoxyadenosine-5′-triphosphate
ATPγS: adenosine 5′-*O*-(thiotriphosphate).
